# 

**Published:** 1891-12

**Authors:** 


### CONTENTS.

COMMUNICATIONS.

The Genesis of the Contour Filling........ 577
The Teeth of Invertebrate Animals........ 587
Mouth Breathing not the cause of Contracted Jaws and High Vault.. 594
Stomatitis........ 605
An Ossified Man Broken........ 616
The Michigan Dental Law........ 617

PROCEEDINGS.

Missouri State Dental Association........ 620

EDITORIAL.

Bogus Medical Colleges........ 621
Again in Harness........ 623
An Appointment Book........ 624
Bibliography........ 624
Obituary........ 625

